# Revisiting insulin-stimulated hydrogen peroxide dynamics reveals a cytosolic reductive shift in skeletal muscle

**DOI:** 10.1016/j.redox.2025.103607

**Published:** 2025-03-25

**Authors:** Carlos Henríquez-Olguín, Samantha Gallero, Anita Reddy, Kaspar W. Persson, Farina L. Schlabs, Christian T. Voldstedlund, Gintare Valentinaviciute, Roberto Meneses-Valdés, Casper M. Sigvardsen, Bente Kiens, Edward T. Chouchani, Erik A. Richter, Thomas E. Jensen

**Affiliations:** a August Krogh Section for Human and Molecular Physiology, Department of Nutrition, Exercise and Sports, Faculty of Science, University of Copenhagen, Denmark; b Exercise Science Laboratory, Faculty of Medicine, Universidad Finis Terrae, Av. Pedro de Valdivia 1509, Santiago, Chile; c Department of Cell Biology, Harvard Medical School, Boston, MA, USA; d Department of Cancer Biology, Dana-Farber Cancer Institute, Boston, MA, USA

## Abstract

The intracellular redox state is crucial for insulin responses in peripheral tissues. Despite the longstanding belief that insulin signaling increases hydrogen peroxide (H_2_O_2_) production leading to reversible oxidation of cysteine thiols, evidence is inconsistent and rarely involves human tissues. In this study, we systematically investigated insulin-dependent changes in subcellular H_2_O_2_ levels and reversible cysteine modifications across mouse and human skeletal muscle models. Utilizing advanced redox tools—including genetically encoded H_2_O_2_ sensors and non-reducing immunoblotting—we consistently observed no increase in subcellular H_2_O_2_ levels following insulin stimulation. Instead, stoichiometric cysteine proteome analyses revealed a selective pro-reductive shift in cysteine modifications affecting insulin transduction related proteins, including Cys179 on GSK3β and Cys416 on Ras and Rab Interactor 2 (RIN2). Our findings challenge the prevailing notion that insulin promotes H_2_O_2_ generation in skeletal muscle and suggest that an insulin-stimulated pro-reductive shift modulates certain aspects of insulin signal transduction.

## 1 Introduction

Insulin, an essential endocrine regulator of post-prandial anabolism, classically stimulates intracellular phospho-signaling downstream of the insulin receptor, a process that has long been proposed to be modulated by generation of reactive oxygen species (ROS)[[1], [2], [3], [4]]. This is proposed to occur via selective, reversible sulfenylation of cysteines in proteins including phosphatase and kinases, triggered by localized production of hydrogen peroxide (H_2_O_2_) by NADPH oxidases and/or mitochondria [[1], [2], [3], [4]]. However, the evidence supporting insulin-induced H_2_O_2_ generation is inconsistent, often derived from non-specific methodologies, and frequently confuses the effect of insulin-stimulated H_2_O_2_ production with the insulin-mimetic or modifying proprieties of H_2_O_2_ [1]. Also, our recent work showed normal glucose tolerance, insulin-responsiveness and skeletal muscle insulin signaling in chow-fed *Ncf1∗* mice lacking NOX2 activity [5], suggesting that NOX2 is not the source of insulin-stimulated H_2_O_2_ *in vivo*, inconsistent with previous reports in cultured muscle cells [6,7]. Due to these discrepancies, our study aimed to study insulin-stimulated H_2_O_2_ dynamics using state-of-the-art compartment-specific redox tools in mouse and human muscle across *in vivo* and *in vitro* conditions.

## 2 Results and discussion

**Fig. 1:**
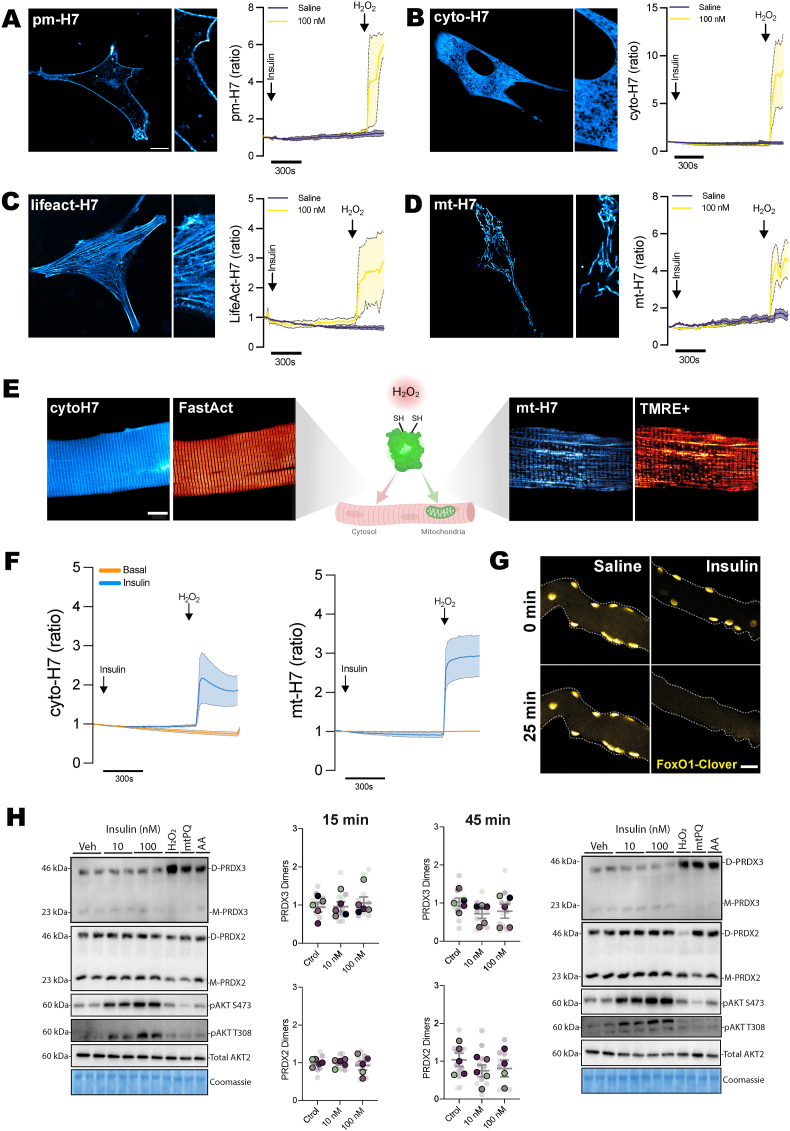
**Insulin does not increase H_2_O_2_ concentration in mouse myoblast and mature muscle fibers.** C2C12 mouse myoblasts were transfected with HyPer7 (H7) probes targeted to (A) the plasma membrane, (B) cytosol, (C) actin cytoskeleton, and (D) mitochondrial matrix. (E) Mouse flexor digitorum brevis (FDB) muscles were electroporated with (F) cytosol-targeted H7, mitochondrial-targeted H7, and (G) FoxO1-clover. Following electroporation, muscle fibers were isolated and cultured. Live imaging was performed by stimulating muscle cells with 100 nM insulin or saline solution for 30 min. (H) 2-Cys peroxiredoxins (PRDX) oxidation and insulin signaling in 7-day differentiated C2C12 myotubes after 10 nM and 100 nM insulin stimulation (n = 5). In myoblasts (A–D), 10 cells were evaluated across three separate experiments, and for muscle fibers (F–H), 6–10 fibers from three different mice were imaged. 50 μM H_2_O_2_ for 30 s, 10 μM Antimycin A (AA) for 2 h, and 10 μM mitochondria-targeted paraquat (mtPQ) were used as positive controls. Scale bar = 10 μm.

Previous studies suggest that insulin-stimulated H_2_O_2_ generation stems from cell surface and endosome-embedded NADPH oxidase 2 complex (NOX2) or endoplasmic reticulum NOX4 [[1], [2], [3], [4]]. Therefore, we hypothesized that H_2_O_2_ generation occurs locally at the plasma membrane and within the cytosol downstream of the insulin receptor. We expressed the ultrasensitive H_2_O_2_-specific biosensor, HyPer7 (H7), which is reported to be pH stable, ratiometric, and sensitive enough to detect fluctuations around the resting low nM and even sub-nM cytosolic H_2_O_2_ concentration [8]. We expressed H7 in myoblasts, targeting subcellular microdomains such as the sarcolemma (Fig. 1A), cytosol (Fig. 1B), and actin cytoskeleton (Fig. 1C) to comprehensively monitor real-time changes in H_2_O_2_ levels within these locations. Despite strong activation of Akt signaling in response to insulin (Fig. 1G and H), we observed no detectable changes in H_2_O_2_ in these compartments, neither in myoblast (Fig. 1A–C) nor in adult muscle fibers (Fig. 1F).

We also expressed H7 targeted to the mitochondrial matrix, as previous studies have suggested glucose-mediated mitochondrial H_2_O_2_ production in this compartment (11, 12). However, the mitochondrial targeted H7 probe did not respond to super-physiological 100 nM insulin treatment during ∼20 min of live-cell imaging in skeletal myoblast (Fig. 1D) or adult muscle fibers (Fig. 1F). Importantly, we replicated previous observations [6,9] in muscle fibers of insulin-stimulated oxidation of 2′,7′-dichlorodihydrofluorescein (DCFH) (Fig. S1A), a widely used unspecific method to measure ROS [10]. Taken together, insulin-stimulated H_2_O_2_ generation is undetectable by the ultrasensitive H7 probes in cultured myoblast and mature muscle fibers, indicating that previously observed DCFH changes are unlikely to be H_2_O_2_-driven.

**Fig. 2:**
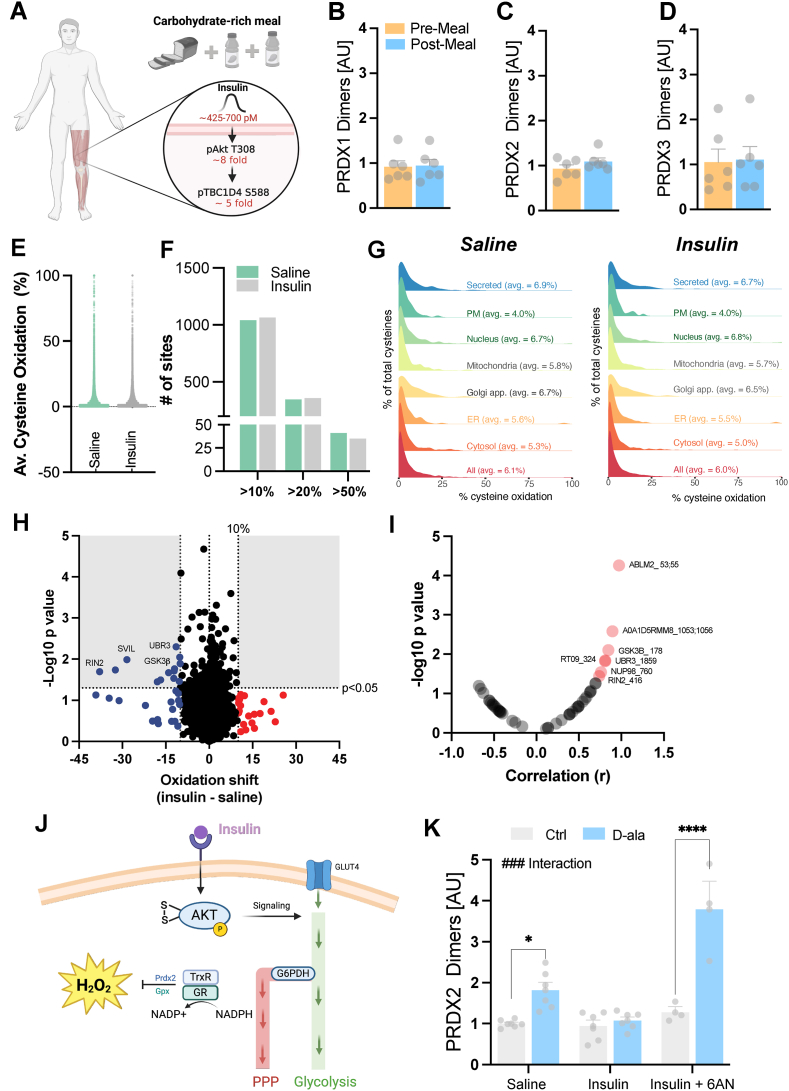
**Insulin promotes a reductive oxidative shift in cysteine proteome**. (A) Vastus lateralis muscle biopsies from six healthy young males were obtained before and after a carbohydrate-rich meal. Non-reducing western blotting was performed to detect dimeric and monomeric forms of 2-cys peroxiredoxins (PRDX): (B) PRDX1, (C) PRDX2, and (D) PRDX3. Data are presented as individual values and means; repeated measures two-way ANOVA was performed. (E) Average percentage and (F) distribution of the cysteine proteome in saline- and insulin-injected mice (n = 4). (G) Percent oxidation distribution of the cysteine proteome across subcellular compartments under saline and insulin treatments. (H) Pairwise comparison of cysteine modification states between saline and insulin treatments. (I) Correlation between blood glucose levels after insulin stimulation and changes in the percentage of cysteine oxidation. (J) Experimental design using chemogenetics and the pentose phosphate pathway (PPP) inhibitor 6-Aminonicotinamide (6-AN). (K) PRDX2 dimerization in response to d-alanine and 6-AN.

Since the insulin-stimulated H_2_O_2_ model has not been tested in humans, we evaluated endogenous compartment-specific H_2_O_2_ changes with insulin by measuring the oxidation state of 2-Cys Peroxiredoxin (PRDX) [11]. In brief, the 2-Cys PRDX isoforms are localized to different subcellular compartments, where they form dimers upon reversible sulfenylation at low H_2_O_2_ concentrations, whereas irreversible sulfonylation prevents dimer formation at high H_2_O_2_ concentrations [12]. Notably, the oxidation of 2-Cys PRDX has been detected at physiological subcellular H_2_O_2_ fluctuations in insects, rodents, and human cells [[13], [14], [15]]. We evaluated *in vivo* H_2_O_2_-mediated PRDX oxidation in human skeletal muscle under fasting and post-prandial conditions. *Vastus lateralis* muscle biopsies were taken before and 90 min into a carbohydrate-rich meal test, eliciting peak physiological insulin concentrations of ∼700 pM at 45 min and ∼425 pM at 90 min (Fig. 2A) [16] Neither cytosolic PRDX1 and PRDX2 nor mitochondrial matrix PRDX3 responded to endogenous insulin (Fig. 2B–D) despite the robust increase in insulin-stimulated glucose uptake [16]. Consistent with this result, PRDX2 and 3 dimerization was unaffected by different insulin stimulation doses and times in mouse C2C12 myotubes (Fig. 1H), human primary myotubes (Fig. S2A), and in mouse skeletal muscle or white adipose tissue after a retro-orbital insulin injection (Figs. S2D–F). Our positive controls for PRDX responsiveness to H_2_O_2_ and for the insulin effect clearly increased in both cell and adult muscle tissue models (Fig. S2G and Fig. S3). Together, we found no evidence of *in vivo* muscle H_2_O_2_ elevation after insulin stimulation in lean mice or in the postprandial state in healthy young humans.

Cysteine oxidation in insulin signaling proteins has been suggested to modulate protein phosphorylation within the insulin signaling cascade [[1], [2], [3], [4]] To investigate whether insulin stimulation directly promotes reversible cysteine modifications *in vivo*, we employed a quantitative cysteine redox proteomics workflow [17]on quadriceps muscle from mice injected with saline vs. insulin (0.4 U/kg) via retro-orbital injection. For the 5495 cysteine sites quantified, no differences were observable between saline and insulin in the average oxidation degree of ∼6.0 % (Fig. 2E). Also, no differences were found between subgroups stratified by percentage cysteine oxidation (Fig. 2F) or by subcellular compartment annotations (Fig. 2G), showing that insulin does not cause global oxidative shifts in the redox proteome. Interestingly, when comparing saline vs. insulin-treated groups, insulin evoked a reductive shift, with 13 proteins exhibiting a significant decrease greater than 10 % in cysteine oxidation levels in the insulin group (Fig. 2H). These cysteines included Cys179 on GSK3β, a classical insulin-inhibited kinase (see Table 1). Other cysteines on known insulin action-related proteins, such as Cys416 on Ras and Rab Interactor 2 (RIN2) and Cys450 on Supervillin (SVIL), were also selectively reduced by insulin and correlated with the glucose-lowering effect of insulin (Fig. 2I).

**Table 1:** Protein cysteines exhibiting *in vivo* insulin-stimulated pro-reductive shift in absolute oxidation >10 % in mouse quadriceps muscle.

	Protein	Cysteine Site	Oxidation Shift	Log10 p Value
1	GSK3B	178	−10,0	1,9
2	ABLM2	53; 55	−10,2	2,0
3	UBR1	251	−10,3	1,5
4	UBR3	1859	−11,4	2,3
5	NUMA1	1912	−12,0	1,8
6	SSU72	12	−12,1	1,7
7	MTMR5	1664	−12,2	1,5
8	UBE3C	395	−14,0	1,7
9	RT09	324	−16,8	1,5
10	A0A1D5RMM8	1053; 1056	−17,9	1,4
11	SVIL	450	−28,4	2,0
12	NUP98	760	−32,4	1,7
13	RIN2	416	−37,9	1,7

N = 4.

To verify the reductive effects of insulin, we produced cytosolic H_2_O_2_ using the chemogenetic tool d-amino acid oxidase (DAAO) 30 min before insulin stimulation. Under non-insulin-stimulated conditions, increased cytosolic H_2_O_2_ production triggered PRDX2 dimerization. However, insulin stimulation mitigated this effect, indicating that insulin increased the clearance of H_2_O_2_, thereby causing a cytosolic reductive shift (Fig. 2K). NADPH is required for reduction and function of intracellular antioxidant defense systems such as the glutathione and thioredoxin-peroxiredoxin and cytosolic NADPH generation is coupled to glucose metabolism via the pentose phosphate pathway (PPP) [18]. Suspecting that generation of NADPH via the PPP caused the reductive effect by insulin, we incubated DAAO expressing C2C12 myoblast with 6-Aminonicotinamide (6-AN) to inhibit glucose-6-phosphate dehydrogenase and consequently PPP-dependent NADPH generation (Fig. 2J). We observed that 6-AN prevented the insulin-mediated reductive effect in d-alanine-treated cells (Fig. 2K), suggesting that insulin-stimulated glucose metabolism via the PPP and NADPH production drives the insulin-stimulated reductive shift.

Taken together, our comprehensive investigation in mouse and human skeletal muscle using state-of-the art redox tools challenges the long-standing concept that insulin receptor activation in various cell and tissue-types universally increases H_2_O_2_ concentration and causes a one-sided increase in protein cysteine oxidation state permissive to insulin phospho-signaling. Rather, insulin does not cause detectable increases in H_2_O_2_ using state-of-the-art measurement tools and a handful of proteins in fact lower their oxidation state in response to insulin including the classical insulin-regulated kinase GSK3.

The pro-reductive shift seems dependent on insulin-stimulated glucose metabolism via the PPP, likely by producing NADPH to fuel cytosolic antioxidant defense systems [19,20]. The insulin-stimulated reduction of GSK3 is intriguing since GSK3 was recently proposed as a dysregulated node causing mitochondrial ROS-linked insulin-resistance of GLUT4 translocation and glucose transport in adipocytes [21]. Thus, certain aspects of insulin signal transduction might be modulated by a pro-reductive rather than a pro-oxidative shift, a model deserving further scrutiny. Future studies should also investigate whether insulin resistance interferes with the reductive effect of insulin on these newly identified cysteine sites. Finally, since a NOX2-dependent increase in cytosolic H_2_O_2_ is required for exercise/contraction-stimulated muscle glucose uptake [22] and gene expression [23,24], this raises new questions about the distinct mechanisms by which pro-reductive insulin and pro-oxidative exercise regulate muscle glucose uptake and other processes. Understanding these mechanisms could provide new insights into metabolic regulation and diseases.

## 3 Material and methods

### 3.1 Cell culture

C2C12 mouse myoblasts originally generated from Dr. Nobuharu L. Fujiís lab (Tokyo Metropolitan University, Japan) were kindly provided by Prof. Amira Klip (SickKids hospital, Toronto, Canada). Muscle cells were maintained in a culture medium consisting of high-glucose Dulbecco's Modified Eagle's Medium (DMEM) (Cat. 11995073; Thermo Fisher) supplemented with 10 % fetal bovine serum (FBS) (F0804; Sigma Aldrich) and 1 % penicillin-streptomycin (PS) (Cat. 15140122; Thermo Fisher) at 37 °C and 5 % CO_2_ humidified atmosphere. Cell differentiation into myotubes was initiated by switching to a differentiation medium comprising DMEM supplemented with 2 % horse serum (HS) (Cat. 26050-070; Thermo Fisher) and 1 % Antibiotic-Antimycotic. The differentiation medium was replaced every two days during seven days of differentiation.

Myoblasts and 7-day differentiated myotubes maintained on either six or 12-well plates were serum-starved in DMEM for 4 h and subsequently stimulated for 15 or 45 min with either submaximal 10 nM or maximal 100 nM insulin (Actrapid; Novo Nordisk, Bagsværd, Denmark). 50 μM H_2_O_2_ for 30 s, 10 μM Antimycin A (AA) for 2 h, and 10 μM mitochondria-targeted paraquat (mtPQ) were used as positive controls.

### 3.2 Transient transfection of C2C12 muscle cells

C2C12 myoblasts cultured on 35 × 14 mm glass-bottom microwell dishes (P35G-1.5-14-C, MatTek Corporation) and on 6-well plates were replenished with fresh serum-free media medium 1 h before transfection. The cells were then transfected with the H_2_O_2_-sensitive probe H7 targeted to different cellular compartments (9): cytosol (cyto-H7), mitochondrial matrix (mt-H7), plasma membrane (PM-H7) and actin cytoskeleton (LifeAct-H7). The 6 well plates myoblast were transfected with the cytosolic targeted d-amino acid oxidase to manipulate intracellular H_2_O_2_ levels [8]. All transfections were performed following instructions of the JetPRIME transfection reagent (Polyplus transfection, NY, USA). The H7 constructs were kindly provided by Professor Vsevolod V. Belousov (Center for Precision Genome Editing and Genetic Technologies for Biomedicine, Pirogov Russian National Research Medical University, Moscow, Russia).

### 3.3 In vivo gene transfer and single fiber culture

To express H7 biosensors and pLenti-FoxO1-Clover in mature skeletal muscle, *in vivo* electroporation was performed in FDB muscle, as previously described [25]. Female mice 12–14 weeks old were anesthetized by 2–3 % isoflurane inhalation and injected in the foot sole with 10 μL of 0.36 mg/ml hyaluronidase (H3884, Sigma) diluted in Phosphate-Buffered Saline (PBS). After 60 min, 20 μl of 10–20 μg plasmid was injected at the same location. H7 plasmids described above were introduced in muscle fibers for H_2_O_2_ detection. Furthermore, pLenti-FoxO1-Clover, a gift from Peter Rotwein (Addgene plasmid # 67759), was used to confirm insulin-stimulated Akt signaling in live imaging experiments [26].

FDB muscle electroporation was performed by delivering 15 pulses of 20 ms at 1 Hz at an electrical field strength of 75 V/cm using acupuncture needles (0.20 × 25 mm, Tai Chi, Lhasa OMS) connected to an ECM 830 BTX electroporator (BTX Harvard Apparatus). After the procedure, the animals were transferred back to their cages and recovered for at least 7 days.

Mice were sacrificed by cervical dislocation, and FDB was removed under a dissection microscope. Isolated live muscle fibers were obtained by enzymatic digestion of the whole muscle in α-MEM (22571-020, Gibco) containing 2.5 mg/ml collagenase type 1 from clostridium histolyticum (C0130, Sigma-Aldrich) for 90–120 min at 37**°**C on a rotator. After collagenase treatment, individual muscle fibers were obtained by mechanical dissociation using fire-polished Pasteur pipettes. Isolated fibers were seeded overnight in ECM Gel-coated (E1270, Merck) 35 × 14 mm glass-bottom microwell dishes (P35G-1.5-14-C, MatTek Corporation) containing α-MEM supplemented with 10 % fetal bovine serum.

### 3.4 Live imaging procedures

Images were collected using either 25x 0.8 NA or 63x 1.4 NA oil immersion objectives on an LSM 980 confocal microscope (Zeiss) driven by Zeiss Zen Blue. C2C12 myoblast and adult muscle fibers were imaged in the 35 mm glass-bottomed microwell dishes in Krebs Ringer buffer (145 mM NaCl, 5 mM KCl, 1 mM CaCl2, 1 mM MgCl2, 5.6 mM glucose, 10 mM HEPES) while kept at 95 % O2, 5 % CO2 and 37 °C using a heated stage.

The ratiometric H7 was sequentially excited at 405 nm and 488 nm, and emission was collected at 520 nm every 15 s during 20 min. The ratiometric signal of H7 was calculated by first subtracting the background at each wavelength and calculating the ratio by dividing the intensity of the emission signals excited by 488nm/405 nm. FoxO1-clover intensity was excited/collected at 488/520 nm every 15 s for 20 min. For the localization of H7 in muscle fibers, isolated fibers were incubated for 45 minutes at 37°C with either 20 nM tetramethylrhodamine ethyl ester (TMRE+, Life Technologies) to label mitochondria or with SPY650-Fast Act dye (Spirochrome, Switzerland) to label cytosolic actin, following the manufacturer's instructions. After incubation, muscle fibers were washed three times with Krebs Ringer buffer to remove residual dyes prior to imaging. For DCFH oxidation, muscle fibers were incubated for 30 min with 5 μM 2′,7′-dichlorodihydrofluorescein diacetate (ThermoFisher Scientific, USA) diluted in Krebs Ringer buffer. Following incubation, the fibers were washed three times with Krebs Ringer buffer and imaged using 0.2 % laser power by exciting at 488 nm and collecting emissions at 520 nm every 15 s for 20 min.

### 3.5 In vivo insulin stimulation in mice

Skeletal muscle and inguinal white adipose tissue (iWAT) were dissected after a single retro-orbital injection of insulin (0.4 U/kg bodyweight; Actrapid, Novo Nordisk, Denmark) or saline dissolved in Gelofusine (B. Braun, Denmark). Before stimulation, mice were fasted for 4 h from 08:00 a.m. and anesthetized (intraperitoneal injection of 7.5 mg pentobarbital sodium/100 g body weight) for 15 min. Blood samples were collected from the tail vein immediately prior to insulin or saline injection and after 5 and 10 min and analyzed for glucose concentration using a glucometer (Bayer Contour; Bayer, Münchenbuchsee, Switzerland).

### 3.6 Human meal study

Experimental details have been published [16]. In brief, healthy young lean men performed one-legged dynamic knee-extension exercise with one leg while the other leg was resting control. 4 h after exercise, muscle biopsies were obtained from both legs, whereupon a mixed solid meal of 30 kJ per kg body weight was ingested, followed by 2 liquid meals of 20 kJ per kg body weight each at 30 and 60 min after the solid meal. Muscle biopsies were obtained again from both legs 90 min after initiation of the meal. Of the 10 individuals in the experiment, results from the last 6 participants were obtained in the present manuscript. For the sake of clarity, only results from the rested leg are shown since there were no differences between values between the rested and exercised leg.

### 3.7 Primary human myotubes

Primary human skeletal myoblasts (HSKM) obtained from the rectus abdominis muscle of a 17-year-old, non-diabetic male with a body mass index of 27 kg/m^2^ (SK-1111, Donor: P01052-17 M, Cook Myosite) were growth to confluency in Myotonic Basal Media (MB-2222) supplemented with 10 % Myotonic Growth Supplement (MS-3333) and 1 % Antibiotic-Antimycotic (Anti-Anti). L6 and HSKM cells were kept at 37 °C and in a humidified atmosphere of 5 % CO_2_. Differentiation of HSKM into myotubes was initiated by switching to a differentiation medium comprising α-MEM supplemented with 2 % HS (Thermo Fisher Scientific, Cat. 26050-070) and 1 % Anti-Anti. The differentiation medium was replaced every two days during the ten days of differentiation.

### 3.8 Redox proteomics

Stochiometric reversible oxidation of cysteines was performed as previously published [17]. Briefly, following freeze-clamping, 30 mg of quadriceps muscles were then homogenized in ice-cold 20 % trichloroacetic acid (TCA) using TissueLyser II (QIAGEN) at speed setting 30 for 5 min. The lysate from each sample was then split into two identical half-samples containing approximately 200 μg protein each, then washed with 20 % TCA, 10 % TCA, and 5 % TCA twice. One half-sample was resuspended in blocking buffer (100 mM HEPES pH 8.5, 8 M urea, 2 % SDS, 1 mM EDTA, 1 mM DTPA, 10 μM neocuproine, and 35 mM IAM) for 2 h at 37 °C in the dark on a shaking incubator (1500 rpm) to block all unmodified cysteine residues, while the other half sample was treated with labeling buffer (100 mM HEPES, 8 M urea, 2 % SDS, 1 mM EDTA, 1 mM DTPA, 10 μM neocuproine, and 35 mM CPT). Samples were checked every 15 min, and if there are non-soluble proteins at 1 h, spin and remove the pellet. Samples were then digested overnight at 37 °C using trypsin and LysC (1:100 enzyme:protein). A second digestion was performed for 6 h at 37 °C using trypsin (1:100 enzyme:protein). Following protein digestion, 50 μg of peptides were labeled using an 18-plex TMT reagent (Thermo Fisher). A ratio check was performed to ensure even labeling. Samples were desalted and then incubated with 12 μL of lambda protein phosphatase for 2 h at 30 °C. CPT-labeled peptides were then enriched using immobilized metal affinity columns (IMAC). Samples were then fractionated using Pierce High pH Reversed-Phase Peptide Fractionation Kit (Thermo). Following fractionation, samples were desalted using C18 stage tips and resuspended in 5 % formic acid with 5 % acetonitrile. A final amount of 3 μg was used for mass spectrometry analysis.

### 3.9 Protein extraction

Before lysis, cells and adult tissues were incubated with 100 mM N-ethylmaleimide (NEM) (Cat. 23030; Thermo Fisher) in PBS for 10 min on ice to alkylate free cysteines. HSKM and C2C12 myotubes were homogenized in ice-cold lysis RIPA buffer containing 20 mM *β*-glycerophosphate, 10 mM NaF, 2 mM PMSF, 2 mM Na_3_VO_4_, 3 mM Benzamidine, 0.01 mM Aprotinin, 0.01 mM Leupeptin and 100 mM NEM. Each sample was sonicated for 15 s (10 % intensity, Q2000 Qsonica sonicator), and lysate supernatants were collected by centrifugation at 18.320*g* for 10 min at 4 °C. Mouse tissues were homogenized for 1 min at 30 Hz using a TissueLyser II bead mill (Qiagen, USA) in ice-cold homogenization buffer containing: 10 % glycerol, 1 % NP-40, 150 mM NaCl, 50 mM HEPES (pH 7.5), 20 mM sodium pyrophosphate, 10 mM NaF, 20 mM β-glycerophosphate, 2 mM phenylmethylsulfonyl fluoride, 1 mM EDTA (pH 8.0), 1 mM EGTA (pH 8.0), 2 mM Na3VO4, 10 μg/mL leupeptin, 10 μg/mL aprotinin, 3 mM benzamidine and 100 mM NEM. Following end-over-end rotation for 10 min at 4 °C, the samples were centrifuged at 18.320*g* for 20 min, and supernatants were collected and stored at −80 °C. Protein concentration was determined using Pierce™ BCA Protein Assay Kit (#23225; Thermo Fisher). Lysates were then diluted to equal protein concentration using MilliQ H_2_O and sample buffer (62.5 mM Tris pH 6.8, 2 % SDS, 10 % glycerol, 0.01 % bromophenol blue).

### 3.10 SDS-PAGE and western blots analyses

Western blot analyses were performed as previously described [22]. Equivalent amounts of protein from each sample were subjected to 12–15 % SDS-PAGE and semi-dry transferred to polyvinylidene difluoride (PVDF) membranes (Immobilon®-P Transfer Membranes; Millipore). The membranes were blocked in 3 % bovine serum albumin (BSA) for 60 min at room temperature, followed by overnight incubation with the indicated primary antibody at 4 °C. The next day, membranes were incubated with the corresponding secondary antibodies in a 1:7500 dilution in 3 % milk for 60 min at room temperature. After incubation, the membranes were washed 3 times for 5 min in Tris-Buffered Saline with Tween 20 (TBS-T) and then visualized using enhanced chemiluminescence (ECL+, Amersham Biosciences) and ChemiDoc^TM^MP Imaging System (Bio-Rad, USA). After development, the membranes were washed with TBS-T and stained with Coomassie Brilliant Blue to verify even transfer and similar total protein loading. The primary antibodies used in the study are listed in Table 2.

**Table 2:** Antibody information.

Antibody	Source	Dilution	Catalog (#)
Akt1/2 Ser473/474	Cell Signaling Technology	1:1000, 3 % BSA	4051
Akt1/2 Thr308/309	Cell Signaling Technology	1:1000, 3 % BSA	9275
Akt2	Cell Signaling Technology	1:1000, 3 % skim milk	3063
TBC1D4 Thr642	Cell Signaling Technology	1:1000, 3 % skim milk	4288
PRDX3	Abfrontier	1:1000, 3 % BSA	PA0030
PRDX2	Abcam	1:1000, 3 % BSA	109367

### 3.11 Statistical analyses

Data are expressed as mean ± SEM with individual data points (when applicable) and analyzed using GraphPad Prism 8. As appropriate, statistical tests were performed using paired/non-paired t-tests or repeated/non-repeated two-way ANOVA. Tukey's *post hoc* test was performed when ANOVA revealed significant main effects. The significance level was established at p < 0.05.

## CRediT authorship contribution statement

**Carlos Henríquez-Olguín:** Writing – review & editing, Writing – original draft, Visualization, Validation, Supervision, Project administration, Investigation, Formal analysis, Data curation, Conceptualization. **Samantha Gallero:** Writing – review & editing, Investigation, Formal analysis. **Anita Reddy:** Writing – review & editing, Visualization, Investigation, Formal analysis. **Kaspar W. Persson:** Writing – review & editing, Investigation. **Farina L. Schlabs:** Writing – review & editing, Investigation. **Christian T. Voldstedlund:** Writing – review & editing, Investigation. **Gintare Valentinaviciute:** Writing – review & editing, Investigation. **Roberto Meneses-Valdés:** Writing – review & editing, Investigation. **Casper M. Sigvardsen:** Writing – review & editing, Investigation. **Bente Kiens:** Resources, Methodology. **Edward T. Chouchani:** Writing – review & editing, Resources, Methodology, Investigation. **Erik A. Richter:** Writing – review & editing, Resources, Methodology, Investigation, Funding acquisition. **Thomas E. Jensen:** Writing – review & editing, Writing – original draft, Supervision, Resources, Project administration, Funding acquisition, Data curation, Conceptualization.

## Declaration of competing interest

The authors declare that they have no known competing financial interests or personal relationships that could have appeared to influence the work reported in this paper.

## Data Availability

Data will be made available on request.
