# Supplementary figures and images for: Revisiting insulin-stimulated hydrogen peroxide dynamics reveals a cytosolic reductive shift in skeletal muscle
Source: Redox Biol. 2025 Mar 25;82:103607. doi: 10.1016/j.redox.2025.103607 (PMC12001130; doi:10.1016/j.redox.2025.103607)

# Figure S1

**A**

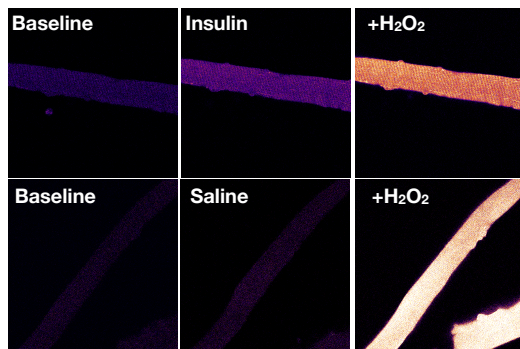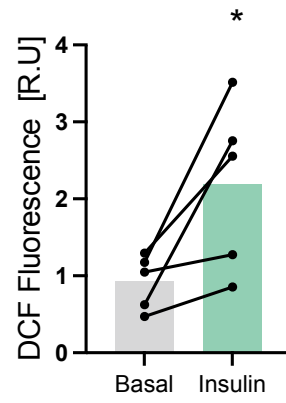

Supplement: Multimedia component 1 [file mmc1.pdf]

Figure S2

A Primary Human Myotubes

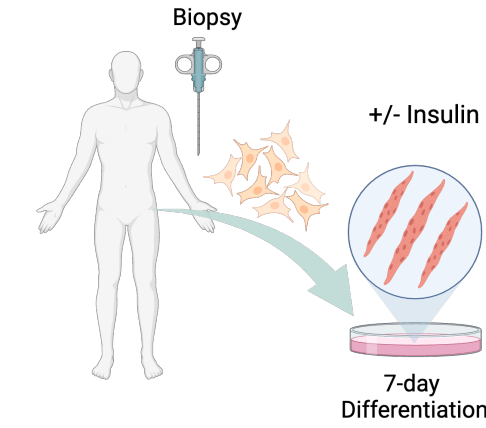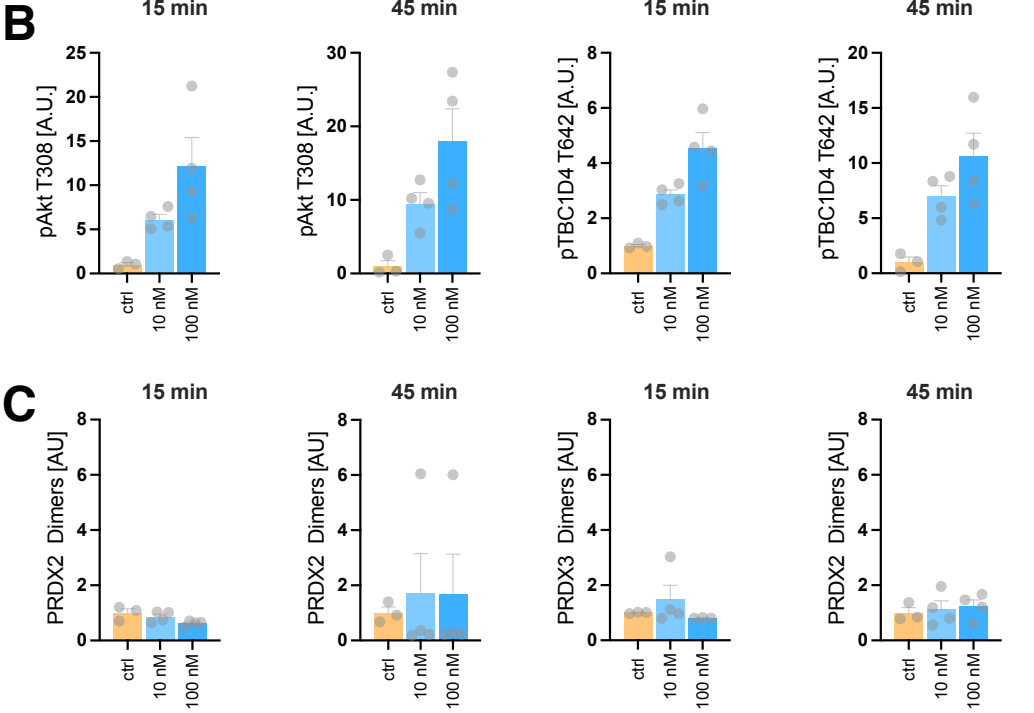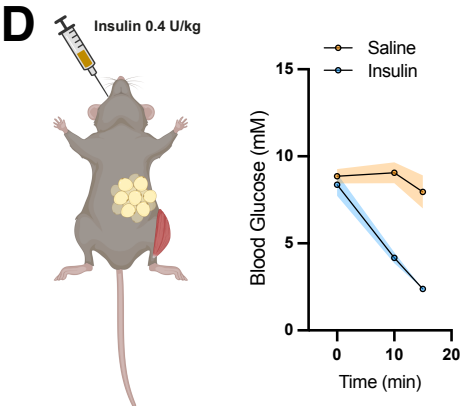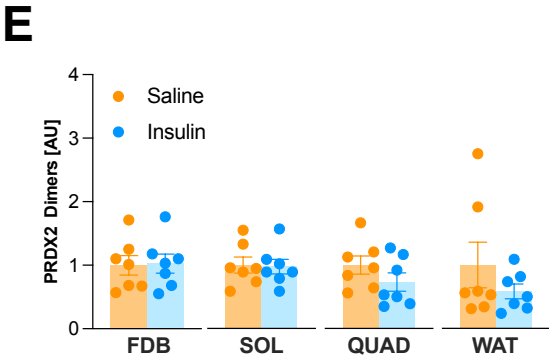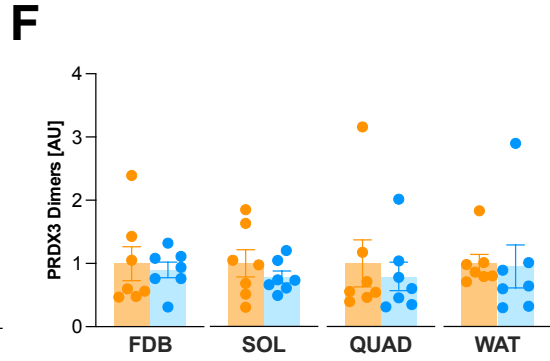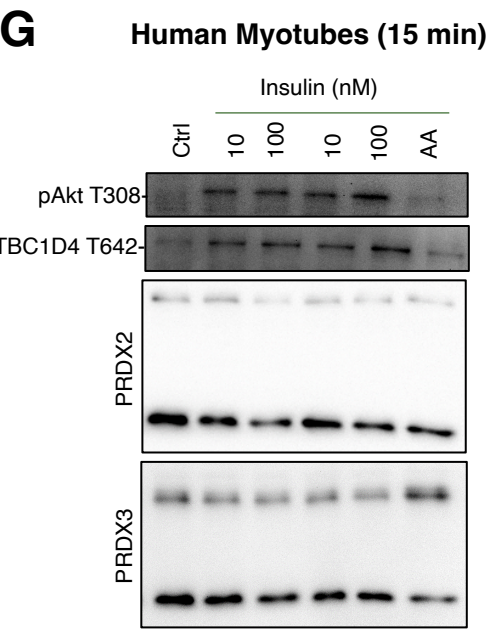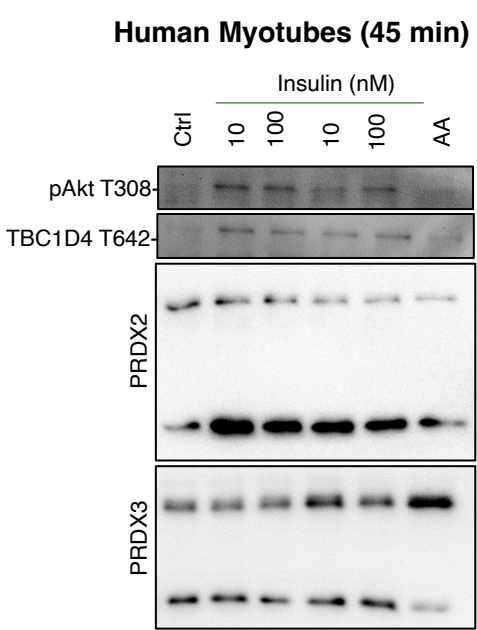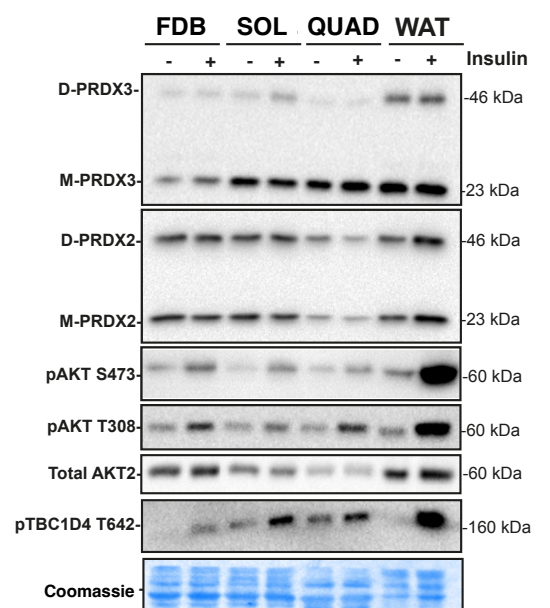

Supplement: Multimedia component 2 [file mmc2.pdf]

Figure S3

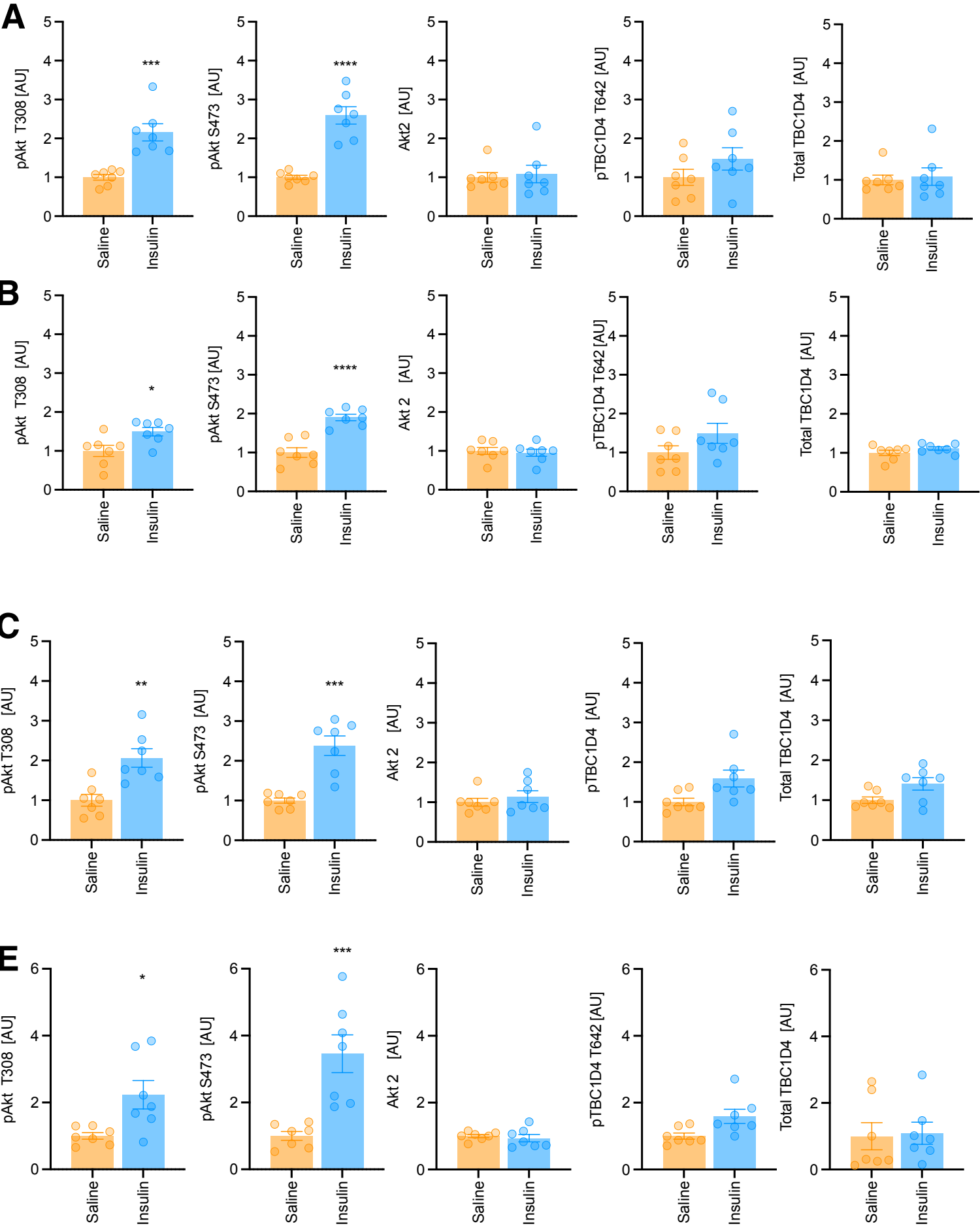

Supplement: Multimedia component 3 [file mmc3.pdf]
